# Association of Blood Pressure and Hypertension with Alcohol Consumption in HIV-Infected White and Nonwhite Patients

**DOI:** 10.1155/2013/169825

**Published:** 2013-10-21

**Authors:** Maria Leticia R. Ikeda, Nêmora T. Barcellos, Paulo R. Alencastro, Fernando H. Wolff, Ajácio B. M. Brandão, Flávio D. Fuchs, Sandra C. Fuchs

**Affiliations:** ^1^Postgraduate Program in Cardiology, School of Medicine, Universidade Federal do Rio Grande do Sul, R. Ramiro Barcelos 2600, 90035-003 Porto Alegre, RS, Brazil; ^2^Hospital Sanatório Partenon, Health State Department of Rio Grande do Sul 3722, Avenida Bento Gonçalves, 90650-001 Porto Alegre, RS, Brazil; ^3^Postgraduate Program in Epidemiology, School of Medicine, Universidade Federal do Rio Grande do Sul, R. Ramiro Barcelos 2600, 90035-003 Porto Alegre, RS, Brazil; ^4^National Institute for Health Technology Assessment (IATS/CNPq), Clinical Research Center, Hospital de Clinicas de Porto Alegre, R. Ramiro Barcelos 2350, 90035-003 Porto Alegre, RS, Brazil

## Abstract

*Introduction.* Although alcohol abuse is associated with hypertension in whites and nonwhites, it has been scarcely investigated in HIV-infected patients. *Objective.* To investigate whether the association of alcohol abuse with hypertension is influenced by skin color in HIV-infected individuals. *Methods.* Cross-sectional study in HIV-infected individuals aged 18 years or older. Demographic characteristics, lifestyle, and HIV infection were investigated. Alcohol abuse was defined as ≥15 (women) and ≥30 g/alcohol/day (men), and binge drinking by the intake of ≥5 drinks on a single occasion. Hypertension was defined by blood pressure ≥140/90 mmHg or use of blood pressure-lowering agents. *Results.* We studied 1,240 individuals, with 39.1 ± 10 years, 51% males and 57% whites. Age and body mass index were associated with blood pressure, and there was an independent association of alcohol abuse with hypertension in whites (RR = 1.9, 95% CI 1.1–3.3) and nonwhites (RR = 2.4, 95% CI 1.4 to 4.0). Among nonwhite individuals who were alcohol abusers, systolic (9.3 ± 3.2; *P* = 0.001) and diastolic blood pressures (6.4 ± 2.1; *P* = 0.008) were higher than in nonabusers. *Conclusion.* Alcohol abuse is a risk factor for hypertension in white and nonwhite HIV-infected individuals. The association of ethanol consumption with blood pressure is not explained by AIDS-related conditions.

## 1. Introduction

Hypertension is a major cardiovascular risk factor worldwide. Projections are that, by the year of 2025, 75.0% (or 1.17 billion people) of the people with hypertension in the world will be living in emerging nations [1]. In Brazil, about 29% of the Brazilian population has hypertension [2]. Alcohol abuse is associated with elevated blood pressure [3], regardless of other risk factors [4–6]. Different studies have shown prevalence rates of hypertension attributed to alcohol consumption ranging from 5 to 30% [7].

Risk factors for cardiovascular diseases, including hypertension and dyslipidemia, are determinants of reduced life expectancy in HIV-infected patients [8]. Hypertension prevalence in HIV-infected individuals ranges from 5.9 to 56.4% [9–12] and has been associated with alcohol abuse and other factors related to HIV infection [9, 10, 13]. In these individuals, the prevalence of alcohol abuse ranges from 8% [14, 15] to 50% [16–18], exceeding the rates in general populations of the United States [19], Europe [20], and Brazil [21], where the prevalence is between 2 and 41% in men and 0.1 and 21% in women [20].

The relationship between alcohol consumption and hypertension may be influenced by some characteristics, such as skin color [3, 22]. Skin color has been identified as a marker of lifestyle [3, 23, 24] and, in some countries, it may also be characterized as the socioeconomic status of the individuals [4]. In Brazil, the HIV-infected population tends to be more homogeneous in terms of socioeconomic status, affecting the less privileged ones. High blood pressure has been associated with low socioeconomic status [4] and skin color may also be a risk factor. Therefore, the objective of the present study was to investigate if the association between alcohol abuse, blood pressure, and hypertension in HIV-infected patients is influenced by skin color.

## 2. Materials and Methods

We conducted a cross-sectional study including HIV-infected individuals who were being followed up at the Outpatient Clinic of the Care and Therapy Service (SAT), Hospital Sanatório Partenon, State Department of Health of Rio Grande do Sul, Brazil. This clinic and all public health care centers provide medical care, antiretroviral therapy (ART), antihypertensives, and other medications free of charge for HIV-infected patients. We consecutively enrolled female and male patients, aged 18 or older, who were seen between June 2006 and December 2008. Pregnant women, individuals who were unable to provide written consent, or those incarcerated were excluded. Participants who were under the influence of alcohol or drugs at the time of the interview were asked to return for evaluation at another time. 

Participants were interviewed on the day of the regular medical appointments. Since they had to consult every month, in order to get antiretroviral medication, they were seen often, which contributed to achieving high participation rate. Information was collected, using standardized questionnaires, for the following characteristics: demographic (age and skin color self-reported, classified as white or nonwhite), socioeconomic (educational attainment, number of school years), and lifestyle characteristics (alcohol consumption, physical activity, and smoking), as well as the use of antiretroviral therapy (ART), and time since diagnosis of HIV infection (in years, classified as ≥6 years, from 3.0 to 5.9 years, and <3 years). Data related to HIV infection as CD4 lymphocyte count (mm^3^/mL), viral load (copies per mL of blood), and clinical information were confirmed by medical records. Alcohol consumption was investigated using a validated quantity-frequency questionnaire based on the kind of beverage consumed [21], administered by a physician or healthcare professional, containing questions about type, frequency, and amount of alcohol consumed in the last six months. Alcohol consumption was categorized into abstemious drinking, social drinking (>0 and <15 grams/day for women and >0 and <30 grams/day for men), or abusive drinking (≥15 grams/day for women and ≥30 grams/day for men) [21, 22, 25]. Binge drinking was characterized by a consumption of five or more drinks on a single occasion [22]. Frequency of consumption was classified as weekly (consumption on some days of the week, but not daily) or monthly (consumption on some days of the month but not every week), regardless of the amount. Physical activity was investigated using the IPAQ (International Physical Activity Questionnaire) [26], which investigates frequency and duration of physical activity. The intensity of exercise was classified as moderate or high versus low physical activity according to the IPAQ protocol [27]. Smoking was defined according to the information provided by the participants, classifying the amount of consumption in at least 100 cigarettes during their lifetimes. 

Weight (Kg) and height (m) were measured twice with the participants being barefoot and wearing light clothes, using an anthropometric scale (Filizola, model 31 adult, Indústria Filizola S.A., São Paulo, SP, Brazil). Body mass index (BMI; kg/m^2^) was calculated as the weight (kg) divided by the square of the height (m) and classified as normal (<25.0), overweight (25–29.9), and obesity (≥30.0). Standardized measurements of blood pressure were obtained in duplicate in two visits, using a validated oscillometric device (OMRON CP-705) [28]. The average of four BP measurements was used to detect hypertension if systolic blood pressure ≥140 mmHg or diastolic blood pressure ≥90 mmHg, or in use of blood pressure-lowering agents [25]. 

Certified researchers performed the interviews and measured anthropometric parameters and blood pressure. Approximately 5% of the interviews were repeated for quality control. Data were entered twice into the database of Epinfo, version 2000. The study was approved by the Research Ethics Committee of the Hospital de Clínicas de Porto Alegre, which is accredited by the Office of Human Research Protections. All participants signed an informed consent form. 

*Sample Size Calculation and Statistical Analysis*. Sample size calculation was based on the estimated prevalence of hypertension between white and nonwhite participants with abusive alcohol consumption (30 and 35%, resp.) and abstemious drinking (18%), with 80% of power and a significance level of 0.05 (two-tailed). A sample size of 738 white and 398 nonwhite participants with a 1 : 5 ratio for exposed and unexposed participants to excessive alcohol consumption would be required to detect a hazard ratio of at least 1.7. The estimates were based on previous studies [22, 29].

Characteristics of the sample were expressed as mean and standard deviation. We used Pearson's chi-square test for the analysis of categorical variables and analysis of variance (continuous variables) to assess the association between these factors and the different outcomes. Confounding factors were controlled using the modified Poisson regression (robust variance estimates) [30] and analysis of covariance. Statistical analyses were performed using the Statistical Package for the Social Sciences (SPSS Inc., Chicago, Illinois, USA), version 18.0. Confounding factors were selected based on the literature [3, 31, 32] and *P* values lower than or equal to 0.2 in unadjusted analysis. Age, sex, educational attainment, smoking, physical activity, body mass index, time since HIV infection, and lifetime HAART use were considered confounding factors used in the multivariate models for whites and nonwhites. Partially adjusted models of alcohol consumption with systolic or diastolic blood pressure, including age, sex, and educational attainment, were run in order to better understand the associations and to interpret the risk ratios. A trend for association was determined by *P* values between 0.05 and 0.1.

## 3. Results

Of the 1,295 HIV-infected patients screened, 1,255 were eligible and 1,240 were included. Thus, forty patients had not confirmed eligibility due to age under 18 years, incarceration, or pregnancy; fifteen refused to participate, and two patients were included in a second attempt due to alcohol intoxication at the first visit. Characteristics of the overall sample are presented in Table 1 by skin color. Participants were aged 39.1 ± 10, half of them were males, and 57% had white skin color. Overall, the distribution of most characteristics was similar among whites and nonwhites, but whites had higher education and time since HIV infection, and nonwhites had high physical activity and prevalence of hypertension.

Risk factors for hypertension among whites and nonwhites are shown in Table 2. The unadjusted analysis showed that only age and BMI were positively associated with hypertension for both categories of skin color. White smokers had lower prevalence of hypertension, whereas among nonwhites there was a trend for the association of alcohol abuse with hypertension. There was no association between variables related to HIV and hypertension.

Table 3 shows that association among the different patterns of alcoholic beverages consumption is roughly similar in whites and nonwhites; abusive consumption of alcohol was associated with hypertension in white and nonwhite HIV-infected individuals, independently of a large set of confounding variables. The frequency of alcohol consumption and acute intake of large amounts were not associated with hypertension.

Systolic and diastolic blood pressure was higher exclusively in nonwhite participants who abused alcohol, independently of confounding factors (Table 4). Systolic blood pressure was on average 9.3 ± 3.2 mmHg, and diastolic 6.4 ± 2.1 mmHg greater among abusers. These differences were independent of age, sex, and educational attainment (data not shown) and of full control for confounding factors. Among white participants, there were an association of diastolic blood pressure with weekly consumption of alcoholic beverages in comparison with nondrinkers and a borderline association versus monthly consumers.

## 4. Discussion 

In this study, we found that the consumption of large amounts of alcohol was independently associated with hypertension in white and nonwhite HIV-infected individuals. Blood pressure, on the other hand, was higher exclusively in nonwhite abusers of ethanol, demonstrated after adjustment for confounding factors. However, even partially adjusted models showed the presence of negative confounding factors. Among, whites there was an association of blood pressure with the frequency of consumption. Taken together, these findings suggest that among nonwhite participants quantity is more important than the pattern of consumption, while for whites the frequency of drinking is more relevant.

The association between alcohol abuse and hypertension identified in our study is in agreement with findings from other populations [33]. The association of blood pressure with abusive consumption exclusively in nonwhites was similar to the observed one in the ARIC cohort study (Atherosclerosis Risk in Communities) [3] and in a cohort carried out in southern Brazil [22]. It is unlikely that this differential association of alcohol abuse and blood pressure by skin color is due to race or ethnicity, but probably relies on other behavioral characteristics of nonwhite individuals [34]. Anyway, blood pressure was higher exclusively in nonwhite abusers, suggesting that these individuals are at higher risk of the harmful effects of ethanol.

Our study adds a piece of mind in the investigation of the influence of HIV-related characteristics and higher incidence of cardiovascular disease. It has been suggested that the use of HAART may be associated with increasing blood pressure [9, 10, 35, 36]. In some studies, longer time of HAART was associated with hypertension [13, 37]. These associations were not identified in our study, since the use of antiretrovirals, time of HIV infection, AIDS, and CD4 count were not associated with hypertension. 

Some limitations of our study deserve to be mentioned. The cross-sectional design does not allow inferring causality. Data on alcohol consumption were obtained from a standardized questionnaire containing detailed information, but it relies on memory, and because it was applied in the context of medical care, this might have led individuals to underreport consumption. Another aspect to be considered is the fact that blood pressure measurements were performed at the medical consultation, and therefore the possibility of a white coat effect cannot be discarded. Even underreporting alcohol consumption or the white coat effect, the association of alcohol abuse with hypertension has been confirmed by ambulatory monitoring of blood pressure [38]. The prospective planning, together with the extensive and detailed measurement of AIDS-related and nonrelated characteristics and adequate statistical power, is a strength of our investigation.

## 5. Conclusions 

In conclusion, alcohol abuse is associated with increased risk of hypertension in white and nonwhite HIV-infected individuals. Blood pressure is higher only in nonwhite individuals who abuse alcoholic beverages and in whites who drink weekly. The association of ethanol consumption with blood pressure is not explained by AIDS-related conditions.

## Figures and Tables

**Table 1:** Characteristics of HIV-infected individuals according to skin color (*n* (%) or mean ± SD).

	Total (*n* = 1240)	Whites (*n* = 710)	Nonwhites (*n* = 530)	*P* value
Male	628 (50.6)	391 (55.1)	237 (44.7)	<0.001
Age (years)	39.1 ± 10.0	39.3 ± 10.3	38.9 ± 9.7	0.5
Educational attainment (years)	7.5 ± 4.1	8.3 ± 4.2	6.4 ± 3.7	<0.001
Smoking	525 (42.3)	298 (42.0)	227 (42.8)	0.4
Physical activity				0.049
Low	321 (25.9)	199 (28.0)	122 (23.0)	
Moderate	398 (32.1)	232 (32.7)	166 (31.3)	
High	521 (42.0)	279 (39.3)	242 (45.7)	
Alcohol consumption				0.7
Abstemious	414 (33.4)	233 (32.8)	181 (34.2)	
Social drinking	757 (61.0)	440 (62.0)	317 (59.8)	
Abusive drinking	69 (5.6)	37 (5.2)	32 (6.0)	
Frequency of alcohol consumption				0.7
Abstemious	414 (33.4)	233 (32.8)	181 (34.2)	
Monthly	440 (35.5)	249 (35.1)	191 (36.0)	
Weekly	386 (31.1)	228 (32.1)	158 (29.8)	
Binge drinking	211 (17.0)	117 (55.5)	94 (44.5)	0.7
Abstemious	414 (33.4)	233 (32.8)	181 (34.2)	
No	616 (49.7)	360 (50.7)	255 (48.1)	
Yes	210 (16.9)	117 (16.5)	94 (17.7)	
Body mass index (kg/m²)	24.9 ± 4.4	24.8 ± 4.3	25.0 ± 4.7	0.4
Systolic blood pressure (mmHg)	121.8 ± 18.2	121.8 ± 17.7	121.8 ± 18.8	0.9
Diastolic blood pressure (mmHg)	76.6 ± 11.4	76.0 ± 10.7	77.3 ± 12.3	0.06
Hypertension	241 (19.4)	120 (16.9)	121 (22.8)	0.009
Time since HIV infection (years)				0.005
≥6.0	404 (32.6)	250 (35.3)	154 (29.1)	
3.0–5.9	350 (28.2)	176 (24.8)	174 (32.8)	
<3.0	485 (39.1)	283 (39.9)	202 (38.1)	
HIV/HCV coinfection	261 (22.2)	143 (21.2)	118 (23.5)	0.3
AIDS diagnosis	892 (72.2)	510 (72.0)	382 (72.3)	0.9
Lifetime HAART use	815 (65.7)	467 (65.8)	348 (65.7)	0.9
Lifetime protease inhibitor use	468 (37.7)	277 (39.0)	191 (36.0)	0.3
CD4 (cell/mm^3^)				0.08
<350	476 (38.8)	258 (36.7)	218 (41.6)	
≥350	751 (61.2)	445 (63.3)	306 (58.4)	

HAART: highly active antiretroviral therapy; HCV: hepatitis C virus; HIV: human immunodeficiency virus; SD: standard deviation.

**Table 2:** Risk factors for hypertension among HIV-infected individuals by skin color (*n* (%)).

	Whites (*n* = 710)		Nonwhites (*n* = 530)	
	Hypertension (%)	RR (95% CI)	Hypertension (%)	RR (95% CI)
Sex				
Female	48 (15.0)	1.0	65 (22.2)	1.0
Male	72 (18.4)	1.2 (0.9–1.7)	56 (23.6)	1.1 (0.8–1.5)
*P* value		0.2		0.7
Age (years)				
18–34	20 (7.4)	1.0	23 (11.4)	1.0
35–49	56 (16.9)	2.3 (1.4–3.7)	63 (24.3)	2.1 (1.4–3.3)
50–78	44 (40.4)	5.4 (3.4–8.8)	35 (50.7)	4.5 (2.8–7.0)
*P* value		<0.001		<0.001
Educational attainment (years)				
≥9	58 (17.8)	1.0	23 (17.8)	1.0
5–8	38 (16.2)	0.9 (0.6–1.3)	51 (22.2)	1.2 (0.8–1.9)
0–4	24 (16.0)	0.9 (0.6–1.4)	47 (27.5)	1.5 (1.0–2.4)
*P* value		0.8		0.14
Smoking				
No	85 (20.6)	1.0	76 (25.1)	1.0
Yes	35 (11.7)	0.6 (0.4–0.8)	45 (19.8)	0.8 (0.6–1.1)
*P* value		0.002		0.16
Physical activity				
High	39 (14.0)	1.0	48 (19.8)	1.0
Moderate	44 (19.0)	1.4 (0.9–2.0)	38 (22.9)	1.2 (0.8–1.7)
Low	37 (18.6)	1.3 (0.9–2.0)	35 (28.7)	1.4 (1.0–2.1)
*P* value		0.3		0.16
Alcohol consumption				
Abstemious	41 (17.6)	1.0	41 (22.7)	1.0
Social drinking	70 (15.9)	0.9 (0.6–1.3)	68 (21.5)	0.9 (0.7–1.3)
Abusive drinking	9 (24.3)	1.4 (0.7–2.6)	12 (37.5)	1.7 (1.0–2.8)
*P* value		0.4		0.08
Frequency of alcohol consumption				
Abstemious	41 (18.0)	1.0	41 (22.7)	1.0
Monthly	41 (17.6)	0.9 (0.6–1.3)	42 (26.6)	0.9 (0.6–1.3)
Weekly	38 (15.3)	1.1 (0.7–1.5)	79 (21.2)	1.2 (0.8–1.7)
*P* value		0.7		0.3
Binge drinking				
Abstemious	41 (17.6)	1.0	41 (22.7)	1.0
No	60 (16.7)	0.9 (0.7–1.4)	60 (23.4)	1.0
Yes	19 (16.2)	0.9 (0.6–1.5)	20 (21.5)	1.0 (0.6–1.5)
*P* value		0.9		0.8
Body mass index (kg/m²)				
<25.0	36 (8.7)	1.0	45 (15.3)	1.0
25–29.9	58 (26.6)	3.1 (2.1–4.5)	40 (24.7)	1.6 (1.1–2.4)
≥30.0	26 (32.9)	3.8 (2.4–5.9)	36 (49.3)	3.2 (2.3–4.6)
*P* value		<0.001		<0.001
HIV/HCV coinfection				
No	96 (18.0)	1.0	85 (22.1)	1.0
Yes	22 (15.4)	0.9 (0.6–1.3)	29 (24.6)	1.1 (0.8–1.6)
*P* value		0.5		0.6
AIDS diagnosis				
No	29 (14.6)	1.0	36 (24.7)	1.0
Yes	90 (17.6)	0.8 (0.6–1.2)	84 (22.0)	1.1 (0.8–1.6)
*P* value		0.3		0.5
Time since HIV infection (years)				
<3.0	47 (16.6)	1.0	44 (21.8)	1.0
3.0–5.9	23 (13.1)	0.8 (0.5–1.2)	34 (19.5)	0.9 (0.6–1.3)
≥6.0	50 (20.0)	1.2 (0.8–1.7)	43 (27.9)	1.3 (0.9–1.8)
*P* value		0.18		0.17
Lifetime HAART use				
No	34 (14.0)	1.0	46 (25.3)	1.0
Yes	86 (18.4)	1.3 (0.9–1.9)	75 (21.6)	0.9 (0.6–1.2)
*P* value		0.14		0.3
Lifetime protease inhibitor use				
No	77 (22.7)	1.0	68 (15.7)	1.0
Yes	44 (23.0)	1.0 (0.7–1.4)	52 (18.8)	1.2 (0.9–1.7)
*P* value		0.9		0.3
CD4 (cell/mm^3^)				
<350	39 (15.1)	1.0	50 (22.9)	1.0
≥350	80 (18.0)	0.8 (0.6–1.2)	69 (22.5)	1.0 (0.7–1.4)
*P* value		0.3		0.9

HAART: highly active antiretroviral therapy; HCV: hepatitis C virus; HIV: human immunodeficiency virus; RR: risk ratio.

**Table 3:** Association between alcohol consumption and hypertension according to skin color.

	Whites	Nonwhites
	RR (95% CI)*	RR (95% CI)*
Alcohol consumption		
Abstemious	1.0	1.0
Social drinking	1.2 (0.8–1.7)	1.1 (0.8–1.5)
Abusive drinking	1.9 (1.1–3.3)	2.4 (1.4–4.0)
*P* value	0.09	0.004
Frequency of alcohol consumption		
Abstemious	1.0	1.0
Monthly	1.1 (0.7–1.6)	1.0 (0.7–1.5)
Weekly	1.4 (1.0–2.0)	1.3 (0.9–1.9)
*P* value	0.2	0.18
Binge drinking		
Abstemious	1.0	1.0
No	1.2 (0.8–1.7)	1.1 (0.6–1.8)
Yes	1.2 (0.8–1.9)	1.2 (0.8–1.6)
*P* value	0.6	0.7

*RR adjusted for age, sex, educational attainment, smoking, physical activity, BMI, time since diagnosis of HIV infection, and use of highly active antiretroviral therapy.

**Table 4:** Association of systolic and diastolic blood pressure with alcohol consumption by skin color.

Skin color	Alcohol consumption	BP ± SE (mmHg)	Delta of BP ± SE (mmHg)^∗†^	*P* value^∗†^	Delta of BP ± SE (mmHg)^∗∗†^	*P* value^∗∗†^
*Systolic BP (mmHg) *						
Whites	Abstemious	120.5 ± 1.0	—		—	
	Social drinking	122.5 ± 0.7	2.0 ± 1.3	0.3	—	
	Abusive drinking	123.3 ± 2.5	2.8 ± 2.8	0.7	0.9 ± 2.7	1.0
Nonwhites	Abstemious	120.8 ± 1.3	—		—	
	Social drinking	121.6 ± 0.9	0.8 ± 1.6	0.9	—	
	Abusive drinking	130.9 ± 3.0	10.1 ± 3.3	0.007	9.3 ± 3.2	0.01
*Diastolic BP (mmHg) *						
Whites	Abstemious	74.9 ± 0.6	—		—	
	Social drinking	76.6 ± 0.5	1.7 ± 0.8	0.1	—	
	Abusive drinking	77.4 ± 1.6	2.6 ± 1.7	0.4	0.9 ± 1.7	0.9
Nonwhites	Abstemious	76.6 ± 0.9	—		—	
	Social drinking	77.1 ± 0.6	0.5 ± 1.1	0.9	—	
	Abusive drinking	83.4 ± 2.0	6.9 ± 2.2	0.006	6.4 ± 2.1	0.008

	Frequency of alcohol consumption					

*Systolic BP (mmHg) *						
Whites	Abstemious	120.5 ± 1.0	—			
	Monthly	121.6 ± 1.0	1.1 ± 1.4	0.9	—	
	Weekly	123.5 ± 1.0	3.0 ± 1.5	0.4	2.3 ± 1.8	0.5
Nonwhites	Abstemious	120.8 ± 1.3	—			
	Monthly	121.3 ± 1.2	0.5 ± 1.8	0.8	—	
	Weekly	123.6 ± 1.4	2.8 ± 2.0	0.12	1.9 ± 1.4	0.5
*Diastolic BP (mmHg) *						
Whites	Abstemious	74.9 ± 0.6				
	Monthly	75.7 ± 0.6	0.8 ± 0.9	0.8		
	Weekly	77.6 ± 0.6	2.8 ± 0.9	0.008	2.0 ± 0.9	0.079
Nonwhites	Abstemious	76.7 ± 0.9				
	Monthly	77.5 ± 0.8	0.8 ± 1.2	0.9		
	Weekly	77.7 ± 0.9	1.0 ± 1.3	0.8	0.2 ± 1.2	0.9

SE: standard error; *delta in relation to abstemious drinking; **delta in relation to social drinking or monthly consumption; ^†^analyses of covariance adjusted for age, sex, educational attainment, smoking, physical activity, body mass index, time since HIV infection, and lifetime HAART use.
